# DELIVERING BRAIN HEALTH EQUITY TO COMMUNITIES CHALLENGED BY SDOH

**DOI:** 10.1093/geroni/igad104.0274

**Published:** 2023-12-21

**Authors:** Stephanie Monroe, Daphne Delgado, Stipica Mudrazija

**Affiliations:** UsAgainstAlzheimer’s, Washington, District of Columbia, United States; Center for Brain Health Equity, Washington, District of Columbia, United States; Urban Institute, Washington, District of Columbia, United States

## Abstract

Growing evidence suggests that dementia risk can be modified by brain healthy behaviors, including effective management of hypertension, diabetes, and obesity. In fact, the Lancet Commission released new research showing that managing a dozen risk factors could prevent or delay approximately 40 percent of worldwide dementia cases. Under-resourced communities are at a disadvantage in managing these risk factors however, due to systemic inequities in health and education, access to exercise opportunities, and to nutritious food. Social determinants of health (SDOH)—and policies that address them—must be part of the solution to ensuring better brain health for all American families. Ensuring that this hope and opportunity reaches all families regardless of zip code, income, race, or ethnicity will take greater commitment, investment, and strategies centered in health equity in public health and community-based initiatives. UsAgainstAlzheimer’s will share what they have done to a) identify, adapt, and disseminate tailored messages to minority-serving communities and b) better equip providers to establish trust and reach those they seek to serve by: 1) better understanding the barriers, opportunities and challenges to reach and build trust in historically underrepresented populations; (2) developing tools, strategies and messages that resonate with these populations; and (3) showing how place matters when it comes to implementing effective public health strategies.

